# The Impact of Hospice Care Structures on Care Processes: A Retrospective Cohort Study

**DOI:** 10.1177/10499091241228254

**Published:** 2024-01-17

**Authors:** Everlien de Graaf, Matthew Grant, Frederieke van der Baan, Marieke Ausems, Carlo Leget, Saskia Teunissen

**Affiliations:** 1 Centre of Expertise in Palliative Care Utrecht, Julius Center for Health Sciences and Primary Care, 8124 University Medical Centre Utrecht, Utrecht, the Netherlands; 2 Palliative Care Physician, 8106 The Dutch College of General Practitioners, the Netherlands; 3 University of Humanistic Studies, Utrecht, the Netherlands

**Keywords:** hospice, palliative care, care structures, care processes, quality of care

## Abstract

**Background:** Palliative care is subject to substantial variations in care, which may be shaped through adapting the organisational structures through which care is provided. Whilst the goal of these structures is to improve patient care, there is a lack of evidence regarding their effect on care processes and patient outcomes. **Aims:** This study aims to describe the relationship between care structures and the quantity and domains of care processes in hospice care. **Design:** Retrospective cohort study. **Settings/Participants:** Data were collected from Dutch hospice patient’s clinical records and hospice surveys, detailing hospice structures, patient clinical characteristics and care processes. **Results:** 662 patients were included from 42 hospices, mean age 76.1 years. Hospices were categorised according to their care structures - structured clinical documentation and multidisciplinary meetings. Patients receiving care in hospices with structured multidisciplinary meetings had an increased quantity of documented care processes per patient on admission through identification (median 4 vs 3, *P* < .001), medication (2 vs 1, *P* = .004) and non-medication (1 vs 0, *P* < .001) interventions, monitoring (2 vs 1, *P* < .001) and evaluation (0 vs 0, *P* = .014), and prior to death. Similar increases were identified for patients who received care in hospices with structured documentation upon admission, but these changes were not consistent prior to death. **Conclusions:** This study details that the care structures of documentation and multidisciplinary meetings are associated with increased quantity and breadth of documentation of care processes in hospice care. Employing these existing structures may result in improvements in the documentation of patient care processes, and thus better communication around patient care.

## Introduction

Palliative care is the sum of many parts and processes, as patients, family members, and formal and informal caregivers contribute to provide care.^1,2^ For patients, this care may be subject to variability related to the care providers and location of care.^3,4^ Health systems operate within structures that order how care teams function and provide care, in order to minimise variations and improve the quality of care for patients.^5^ The Donabedian^6^ model identifies care as consisting of 3 components: care structures, processes and outcomes.^6-8^ Structures are defined as the conditions under which care is provided, including the material and human resources, and organisation characteristics, such as qualifications of health professions in the hospice team, or structured use of clinical guidelines.^6,9^ Processes describe the sum of actions that contribute to care, including activities relating to diagnosis, treatment, prevention, education, such as taking blood pressure, identifying symptoms, prescribing medications, identifying symptoms, and interpersonal communication. Outcomes refer to the results of care and whether these goals were achieved (ie, how many patients have unrelieved symptoms).^6,10,11^ Considerable attention has been focused on measuring care processes and outcomes, as measures of quality end of life care.^8,10,12^ However, care processes and outcomes may ‘lie too far down the line to be efficient detectors of quality problems’, and thus applying specific care structures may be the optimal manner to shape how the processes and outcomes of care.^13^ Whilst employing specific care structures may be ideally placed to address care provision at its ‘root’, there is a lack of evidence to connect these structures with improvements to patient care.^14^

Palliative care is subject to considerable heterogeneity and fragmentation of care structures, as diverse providers from various organisations, echelons, professional groups and informal caregivers act together to engage in care provision.^15^ This is particularly relevant in Dutch hospice care, where approximately 300 hospices (in-patient palliative care units) serve different geographical locations and provide care for over 12000 patients annually. Hospices in the Netherlands are heterogenous, including hospices where care is primarily provided by volunteers, units that operate as part of nursing homes, and independent hospices with specialist-trained palliative care staff, with differences in availability and training of staff, use of clinical guidance, and other structures of care.^3,16^ Previous research has demonstrated that the patient populations accessing these hospices do not differ substantially.^17^

A number of care structures have been advocated as means to improving the quality of palliative care.^18^ When implemented on an organisational level, multidisciplinary meetings have demonstrated improved concordance with guidelines, collaboration with other providers, and facilitate continued learning for health professionals.^19,20^ Guidance recommend that meetings should ideally occur at least fortnightly, have a structured approach, and include health professionals from multiple professions.^19,21^ Structured clinical documentation employed on an organisation level has been highlighted as a further structure to influence care processes, and may improve assessment and communication of patient’s multidimensional care needs.^22,23^ Both these care structures are promoted in the Dutch quality framework for palliative care, however, it is not known how, or if, they influence real-world care provision.^21^

Given the heterogeneity of hospice care internationally, if specific structural changes are to be promoted on a broader level, they should be supported by evidence demonstrating their impact on patient care. The aim of this study was to describe the relationship between care structures and the quantity and domains of care processes in hospice care.

## Methods

### Study Design

A retrospective cohort study was performed using routine care data from a representative sample of Dutch hospices. Reporting is in accordance with the Strengthening the Reporting of Observational Studies in Epidemiology (STROBE) Checklist for observational studies.^24^ This research was reviewed by the institutional review board of the UMC Utrecht (18-373/C) and not considered subject to the Medical Research Involving Human Subjects Act of the Netherlands. In line with the principles of Good Clinical Practice, local consent from hospices was obtained.

### Setting and Population

A random sample of Dutch hospices was collated, representing a cross section of hospice organisations and covering geographic locations throughout The Netherlands.^17^ In each participating hospice, 16 adult patients who received hospice care and died in the hospice in 2017 or 2018 were randomly selected using a random number generator. No exclusion criteria were used to give a broad description of Dutch hospice patients.

### Data

Outcomes were the quantity and dimension of care processes for each patient. Care processes were quantified according to differing steps using Clinical Reasoning as a framework (see Table 1): identification, intervention (non-medication or medication), monitoring, and evaluation.^16^ This framework has been employed previously in observational studies in palliative care to describe and quantify care processes.^25^ These were further characterised by the dimension of care need to which they related: physical, psychological, social or spiritual.^25^ Data were collected for each patient in the first and last 72 hours of admission. In the case of patients with an admission of less than 4 days, only the last 72 hours were recorded. In the Dutch hospice system, almost all patient admissions are for end of life care.^16^

**Table 1.** Care Processes: The Steps of Clinical Reasoning.^16^

Step of Clinical Reasoning	Clinical Process
Identification	Identification of the patient’s care needs
Intervention	Intervening to treat the patient’s care need. This could be through
	- Non-medication treatment	
	- Medication treatment	
Monitoring	Monitoring the patient’s care need and/or response to intervention
Evaluation	Evaluating and adapting care or intervention

Patient clinical records were reviewed by the researchers from January 2019 to June 2021, who were experienced palliative care clinicians (nurses, clinical nurse specialists, general practitioners, and physicians) and entered manually using electronic case report forms in Castor Electronic Data Capture.^26^ Interim analyses were performed on inter-rater differences in the data, with overall good concordance, and those patient records with noticeable variances had this data re-collected.^26^ Data were entered as unknown if clinical documentation was unclear or not recorded for that variable.

An electronic survey was sent to all managers of participating hospices through Survey Monkey at the time of collection of patient records. Items focused on the care structures of the hospice, including numbers of beds and admissions, forms of documentation, multidisciplinary meetings, and involvement of care providers. Two commonly utilised care structures implemented on an organisational level (structured documentation and multidisciplinary meetings) were independent variables. Clinical documentation structures refer to the use of documentation employed to record patient clinical notes throughout the admission. The Life Care Plan is a form of documentation developed for nursing home facilities, and aims to notate patient’s function, quality of life impacts and goals of care throughout admission.^27^ Palliative reasoning was developed in the Dutch hospice setting and aims to document patient multidimensional care needs through structured assessment, communication, and documentation.^28^ Multidisciplinary meetings were assessed according to clinical practice guidance, which recommend that such meetings are structured, occur routinely (weekly or fortnightly), and involve a multidisciplinary team including nurses, doctors, and allied health or spiritual care disciplines.^19,21^

### Data Analyses

Descriptive statistics were used to describe the baseline characteristics of the study population. Patients were stratified according to which care structures (structured documentation and multidisciplinary meetings) were employed in the hospice in which they received care, as identified through the hospice managers’ survey. Medians and inter-quartile ranges were used to describe main outcomes (number of care processes) due to nonparametric distribution, with between group differences tested through the Mann-Whitney-U test for 2 groups, and Kruskal Wallis tests for analyses with 3 groups. *P*-values less than .05 were considered statistically significant. Analyses were performed using Rstudio (Rstudio team, Boston, 2020).

## Results

### Hospices

A total 42 hospices (of 51 hospices, response rate 82%) responded to the survey, providing data on their routine care structures and were included for analysis. The organisational structures of the hospices are described in Table 2.

**Table 2.** Care Structures per Hospice, Classified According to Hospice Type.

		Total
Number of beds – median [IQR]		6 [4-9]
Number of admissions in 2018 per hospice – median [IQR]		69 [52-84]
Which allied health professions are available at hospice^a^	Physiotherapist (%)	17 (45)
	Occupational therapist (%)	12 (32)
	Creative therapist (%)	10 (27)
	Psychologist (%)	10 (27)
	Music therapist (%)	9 (24)
	Dietician (%)	9 (24)
Clinical documentation structure employed	Palliative reasoning	13 (31)
	Life care plan	21 (50)
	Other/None	8 (19)
Multidisciplinary meeting occurrence	Regularly (every 1 or 2 weeks)	29 (69)
	Irregular	10 (24)
	None	3 (7)
Which caregivers participate in multidisciplinary meeting^a^	Nurse (%)	39 (93)
	Doctor (%)	37 (88)
	Social worker/psychologist/chaplain (%)	28 (67)
Is a multidisciplinary meeting structure employed?	Yes (%)	28 (67)
	No (%)	14 (33)
Multidisciplinary meeting following guidance (regular, structured and multidisciplinary)	Yes (%)	21 (50)
	No (%)	21 (50)

^a^more than 1 answer possible.

Documentation structure varied between hospices, with 13 hospices (31%) using palliative reasoning method, 21 (50%) using the life-care plan, and a further 8 hospices (19%) employing other or no structure. These other documentation structures included the Omaha method for 2 hospices, 5 with location-specific documentation, and 1 with no documentation structure.^29^

The majority of hospices (69%) had regular multidisciplinary meetings every one or 2 weeks, and 28 (67%) employed a meeting structure. These meetings routinely involved nurses, doctors and an allied health professional in 93%, 88% and 67% of hospices. Overall, 21 hospices (50%) fulfilled all 3 criteria for structural multidisciplinary meetings.

### Patient Characteristics on Admission

In total, 662 patients from the 42 hospices were included in the study, mean age of 76.1 years, described in Table 3. Ten selected patients were not included due to incomplete records. Malignancy was the main diagnosis for 78% of patients, with cardiovascular disease the most prevalent major comorbidity (40%). 66% of patients were orientated and 33% spent greater than half of the day out of bed or chair.

**Table 3.** Patient Demographic and Clinical Characteristics on Admission.

Number patients		662
Age	Mean (SD)	76.1 (12.3)
Gender – female (%)		343 (52)
Length of admission (days)	Median [IQR]	14 [6-37]
Main diagnosis (%)	Malignancy	519 (78)
	Organ failure	73 (11)
	Neurological disease	31 (5)
	Other	29 (4)
	Unknown	10 (1)
Major comorbidity^a^ (%)	Cardiovascular	268 (40)
	Respiratory	96 (15)
	Cancer (other forms of)	51 (8)
	Neurological/dementia	38 (6)
	Renal	28 (4)
Functional status (%)	<50% in bed/chair	218 (33)
	>50% in bed/chair	191 (28)
	Bed-bound	119 (18)
	Unknown	134 (20)
Mental status - orientated (%)	Yes	436 (66)
	No	138 (21)
	Unknown	88 (13)

^a^more than 1 answer possible.

### Document Structure

In the first 72 hours of admission (see Table 4), patients receiving care in hospices using palliative reasoning were reported to receive significantly more care provision compared to life care plan and none/other across all steps of care provision: identification (median 4 vs 3 and 3 respectively, *P* < .001), non-medication treatment (1 vs 0, 0, *P* < .001), medication treatment (2 vs 0, 0, *P* = .010), monitoring (2 vs 1, 1, *P* = .008), and evaluation (0 vs 0, 0, *P* = .002). This care focused on a broader array of care needs, demonstrating more care processes in all dimensions: the physical (median 8 vs 6, 6, *P* < .001), psychological (1 vs 0, 0, *P* < .001), social (0 vs 0, 0, *P* < .001) and spiritual (0 vs 0, 0, *P* < .001).

**Table 4.** Care Provision – Mean Interventions per Patient During First and Last 72 hours According to Form of Structured Documentation Employed.

	First 72 hours				Last 72 hours			
Type of documentation structure employed	Other N = 112	Palliative reasoning N = 181	Life Care Plan N = 276	*P* Value	Other N = 126	Palliative reasoning N = 208	Life Care Plan N = 328	*P* Value
Care processes – median [IQR]								
Identification	3 [1-5]	4 [2-7]	3 [1-4]	<.001	1 [0-3]	2 [1-4]	1 [0-3]	.014
Non-medication treatment	0 [0-1]	1 [0-3]	0 [0-1]	<.001	0 [0-1]	0 [0-2]	0 [0-1]	.025
Medication treatment	1 [0-3]	2 [0-5]	1 [0-3]	.010	3 [1-4]	3 [1-6]	4 [1-7]	.027
Monitoring	1 [0-3]	2 [0-4]	1 [0-3]	.008	2 [1-4]	3 [1-5]	3 [1-6]	.049
Evaluation	0 [0-1]	0 [0-1]	0 [0-0]	.002	0 [0-.75]	0 [0-1]	0 [0-1]	.233
Domains– median [IQR]								
Physical	6 [2-11]	8 [3-16]	6 [2-10]	<.001	7 [3-11]	8.5 [4-14]	8 [3-14]	.037
Psychological	0 [0-2]	1 [0-3]	0 [0-2]	<.001	0 [0-2]	0 [0-4]	0 [0-3]	.260
Social	0 [0-0]	0 [0-0]	0 [0-0]	<.001	0 [0-0]	0 [0-0]	0 [0-0]	.010
Spiritual	0 [0-0]	0 [0-0]	0 [0-0]	<.001	0 [0-0]	0 [0-0]	0 [0-00]	.063

In the last 72 hours, these differences in care provision were less consistent. Patients in the palliative reasoning group had greater numbers of care provision documented through identification (median 2 vs 1, 1, *P* = .014) and non-medication treatment (0 vs 0, 0, *P* = .025). However, patients whose hospices utilised the life care plan had greater quantity of provision in meditation treatment (median 3 vs 4, 3, *P* = .027) and monitoring (3 vs 3, 2, *P* = .049). The domains of care provision were not consistently different dependant on the form of documentation used.

### Multidisciplinary Meetings

In the first 72 hours, patients in hospices that fulfilled the MDM criteria (see Table 5) were reported to receive greater care provision in identification (median 4 vs 3, *P* < .001), non-medication treatment (1 vs 0, *P* < .001), medication treatment (2 vs 1, *P* = .004), evaluation (0 vs 0, *P* = .014). And monitoring (2 vs 1, *P* < .001). This care focused on a broader array of care needs in all dimensions: the physical (median 7 vs 5, *P* = .001), psychological (0 vs 0, *P* = .004), social (0 vs 0, *P* < .001) and spiritual (0 vs 0, 0, *P* < .001).

**Table 5.** Care Processes in First and Last 72 hours of Admission According to Multidisciplinary Meetings.

	First 72 hours			Last 72 hours		
MDM criteria fulfilled	No N = 275	Yes N = 294	*P* Value	No N = 329	Yes N = 333	*P* Value
Care processes– median [IQR]						
Identification	3 [1-5]	4 [2-6]	<.001	1 [0-3]	2 [1-4]	<.001
Non-medication	0 [0-1]	1 [0-2]	<.001	0 [0-1]	0 [0-1]	.010
Medication	1 [0-3]	2 [0-4]	.004	3 [1-5]	4 [1-7]	.001
Monitoring	1 [0-3]	2 [0-4]	<.001	2 [0-4]	3 [1-6]	<.001
Evaluation	0 [0-0]	0 [0-1]	.014	0 [0-1]	0 [0-1]	.582
Domains – median [IQR]						
Physical	5 [2-10]	7 [3-13]	.001	7 [2-12]	9 [5-14]	<.001
Psychological	0 [0-2]	0 [0-3]	.004	0 [0-2]	0 [0-4]	.002
Social	0 [0-0]	0 [0-0]	<.001	0 [0-0]	0 [0-0]	.187
Spiritual	0 [0-0]	0 [0-0]	<.001	0 [0-0]	0 [0-0]	.021

In the last 72 hours, patients in hospices that met the MDM criteria had increased care provision documented through identification (median 2 vs 1, *P* < .001), non-medication treatment (0 vs 0, *P* = .010), medication treatment (4 vs 3, *P* = .001), and monitoring (3 vs 2, *P* < .001). This increase in care processes was most prominent in the physical (median 9 vs 7, *P* < .001) and psychological (0 vs 0, *P* = .002) dimensions.

## Discussion

### Main Findings of the Study

The results of this study suggest that the use of structured documentation and multidisciplinary meetings is associated with increases in the quantity of documented care processes, which focus on a greater breadth of multidimensional issues. Structured documentation in the form of palliative reasoning demonstrated increases in documented care provision in the first 72 hours after admission, though this effect was less evident in the final 72 hours. This is consistent with our hypothesis, that the use of structure documentation is likely to be of greatest impact at the beginning of admission to improve prompt identification and subsequent treatment. At the end of the admission, the majority of care needs have already been identified and managed.

Employing routine and structured multidisciplinary meetings was associated with increases in the quantity and breadth of care processes throughout the first and last 72 hours. It might be expected that multidisciplinary meetings were more likely to effect care later in the admission after meetings had occurred, as a trigger to document care processes, and when care providers had the opportunity to discuss the care needs of the patient from multiple perspectives, and thus more likely to see evidence of increased care processes in the last 72 hours. This may signify that these interprofessional meetings have an osmotic effect beyond the confines of the meeting, facilitating health professionals to have greater awareness of care needs extending beyond their normal sphere of expertise in their daily practice through regular engagement with the multidisciplinary team, and thus recognise a great range of patient care needs at admission.^20^

### What This Study Adds

A criticism of the integration of routine care structures into palliative care practice is the propensity to medicalise care, and thus the dying process.^30,31^ Medicalisation is defined as a characteristic medical form of interpretation of health, illness and the dying process through the frameworks of pathological disease.^31^ James and Field^32^ argued that hospice care is becoming increasingly professionalised “towards more traditional conceptions of disease and its treatment, to the possible detriment of other ‘softer’ aspects” or holistic care”. The results of this study somewhat refute this claim, as structured approaches to care are associated with greater recording of the patient’s non-physical care needs, particularly when first admitted. It might be expected that this ‘medicalisation’ would result in greater use of medication treatments focused on the physical domain, and whilst this was apparent, these differences were consistent through all steps of processes and domains of care, including non-medication treatments and attention to the patient’s psychological, social and spiritual needs. From a conceptual viewpoint, we would argue that a standardisation of care structures is not equal to a medicalisation of care, but rather structuring the approach to multidimensional care, to facilitate a consistent approach to the care of every patient, to identify and meet their individual care needs in the last months of life.

Palliative care has organically developed in relation to the care needs of patients with life-limiting illnesses worldwide. The structures of hospices have evolved in relation to many local, cultural and sociological influences, and should ideally remain responsive to the local culture in which they are imbedded, which may differ considerably dependant on these factors .^2^ This is a challenge when advocating for particular care structures to be implemented in practice, as they may not always be optimally appropriate to patients, their families, staff, and the approach to care.^31^ If specific care structures are to be promoted, these should ideally be (i) adapted to the context of local hospice practices, and (ii) supported by evidence linking them to improved care provision or outcomes.^14,33^ The benefit of using existing care structures is that relevant expertise regarding their use already exists, which could support implementation into new care settings. From a health policy perspective, the drive to improve the quality of palliative care does not mean that all hospices should become the same. Instead, the unique cultural and organisational basis of each hospice should be promoted whilst additionally seeking to employ care structures that can be practically implemented to facilitate quality and appropriate care for each patient.^34^

### Strengths and Limitations of the Study

The main strengths of this study are numbers and range of patients and hospices included, and the use of detailed data describing real-world care provision. Data collection was undertaken by experienced palliative care clinicians, using a comprehensive strategy for a large cohort of hospice patients. The outcome measure – the type and domain of care process - is aligned with multiple quality indicators of end of life care, such as comprehensive assessment and treatment of symptoms, and broad focus on the psychological, social and spiritual aspects of care.^12,21^

This study employed methods that were judged most suitable to the aims of the project, in order to access the reality of how care is provisioned in hospices on a national level.^26^ Such methods are heavily reliant of the quality of documentation, which may vary in content, forms and structure per setting. The increased number of documented clinical processes identified in the structured documentation cohorts is likely reflective of improved documentation of these processes. Yet, the quality of documentation is intertwined with the clinical processes, as improved communication of patient’s needs is more likely to lead to monitoring and evaluation of the patient’s condition at the end of life.^23^ A further limitation of this study is the lack of patient outcome data. We identified in the pilot for this study that whilst clinical care processes were reliably recorded in hospice clinical documentation, patient outcomes were not well documented, and thus it was decided to focus on the association between structures and care processes.^26^

Retrospective observational studies have limitations in their ability to link cause with outcome, and these results examining relationships between care structure and processes should be interpreted with this qualification foregrounded.^35^ There are many other complex structures that influence palliative care provision that cannot be quantified in this research, such as levels of staff education and professional development, use of clinical guidelines to inform routine care provision, and extent of regular multidisciplinary involvement in patient care.^3^ Further research would ideally examine how these and other structures influence patient outcomes, to fully understand the relationship between care structures and quality of care.

## Conclusions

These results suggest that structured documentation and multidisciplinary meetings is associated with improved documentation, and thus, communication of patient care processes. It details that increased numbers of patients care needs were assessed, treated, monitored, and evaluated in the first and last 72 hours of admission. Importantly, this relationship was evident in each domain of patient care – the physical, psychological, social and spiritual. Care structures are important elements of quality care that shape how palliative care is provisioned, and the quality of care and life experiences by patients and their families. These results describe 2 existing care structures associated with increased documentation of patient care provision that can readily be integrated into practice.
